# Using Qualitative-Electroencephalogram (Q-EEG) Mapping to Aid the Selection of Suitable Areas to Target Repetitive Transcranial Magnetic Stimulation (rTMS) Treatment in a Case of Depression With Comorbid Obsessive Compulsive Disorder (OCD)

**DOI:** 10.1192/bjo.2022.82

**Published:** 2022-06-20

**Authors:** Louay Eltagy, Alex O'Neill-Kerr, Nadia Hristova

**Affiliations:** 1 Birmingham and Solihull Mental Health NHS Foundation Trust, Birmingham, United Kingdom; 2 Transforming Mind Solutions, Northampton, United Kingdom; 3 Bright Brain Centre, London, United Kingdom

## Abstract

**Aims:**

We present the case of SN, a 25-year-old woman with diagnosis of anorexia nervosa, OCD, Generalized Anxiety Disorder (GAD) and depression. She has extensive history of contact with mental health services spanning more than 10 years. She has had 1 inpatient stay in an eating disorders unit lasting more than 6 months. Her treatment included various classes of medications, psychological therapy and social prescribing with little or no benefit. She has been referred to rTMS. The aims of the study are to determine the effect of rTMS in treatment of a patient with depression comorbid with OCD, understand the value of q-EEG in rTMS treatment and to treat OCD symptoms using rTMS guided by QEEG.

**Methods:**

SN had a total of 56 rTMS sessions targeting standard depression and anxiety areas; F3 (left sided excitatory) and F4 (right sided inhibitory). Following this her depression and anxiety improved but her OCD worsened. She then underwent a Q-EEG to be able to understand the physiological cause of her symptoms and suggest meaningful further neuromodulation that is tailored to her. This indicated dysregulation within the default mode network. Spindling beta waves were detected over the posterior electrode suggesting a tendency towards ruminations. There was clear hyperactivity in the supplementary motor area. SN had further 30 rTMS sessions targeting the OCD circuit (FC1 and FC2).

**Results:**

Rating scales showed a reduction in Patient Health Questionnaire-9 (PHQ-9) score from 22 to 14 (36%) in second course compared to an increase of PHQ-9 score from 9 to 15 (66.6%) in first course; indicating an overall 102% improvement in PHQ-9. It also showed reduction of Yale-Brown Obsessive Compulsive Scale (Y-BOCS) in second course from 34 to 8. It was not done in the first course but there was a clinical increase in OCD symptoms following the end of the first course. These results were corroborated clinically.

A repeat q-EEG showed that the areas previously highlighted in red at FC1 and FC2 had now all reverted to green, indicating normal neuronal connectivity.

**Conclusion:**

rTMS can provide timely and adequate response to depression and anxiety especially one that has not responded adequately to medications and psychotherapy. Q-EEG is useful to direct the plan, create a personalized plan and achieve accurate results. The use of q-EEG, whilst useful, should be balanced with other considerations as financial constraints. It should be reserved to patients who have not responded favorably to standard rTMS treatment.

